# Leveraging network analysis to evaluate biomedical named entity recognition tools

**DOI:** 10.1038/s41598-021-93018-w

**Published:** 2021-06-29

**Authors:** Eduardo P. García del Valle, Gerardo Lagunes García, Lucía Prieto Santamaría, Massimiliano Zanin, Ernestina Menasalvas Ruiz, Alejandro Rodríguez-González

**Affiliations:** 1 ETS de Ingenieros Informáticos, Universidad Politécnica de Madrid, Boadilla del Monte, Madrid Spain; 2 Centro de Tecnología Biomédica, ETS Ingenieros Informáticos, Universidad Politécnica de Madrid, Pozuelo de Alarcón, Madrid Spain; 3 Instituto de Física Interdisciplinar y Sistemas Complejos IFISC (CSIC-UIB), Campus UIB, Palma de Mallorca, Spain

**Keywords:** Diseases, Bioinformatics, Scientific data, Medical research, Classification and taxonomy, Literature mining, Network topology

## Abstract

The ever-growing availability of biomedical text sources has resulted in a boost in clinical studies based on their exploitation. Biomedical named-entity recognition (bio-NER) techniques have evolved remarkably in recent years and their application in research is increasingly successful. Still, the disparity of tools and the limited available validation resources are barriers preventing a wider diffusion, especially within clinical practice. We here propose the use of omics data and network analysis as an alternative for the assessment of bio-NER tools. Specifically, our method introduces quality criteria based on edge overlap and community detection. The application of these criteria to four bio-NER solutions yielded comparable results to strategies based on annotated corpora, without suffering from their limitations. Our approach can constitute a guide both for the selection of the best bio-NER tool given a specific task, and for the creation and validation of novel approaches.

## Introduction

Huge volumes of digital textual content are generated every day in biomedical research and practice, including scientific papers, electronic medical records (EMRs), and physician notes. These sources contain information about new discoveries and new insights, providing valuable knowledge for medical applications such as disease–disease relationships or drug repositioning. However, medical texts consist mainly of unstructured, free-form textual content that requires manual curation and analysis performed by domain experts^1^. Since the manual curation and management of such large corpora are infeasible, over the last decades biomedical researchers have relied on natural language processing (NLP) methods and techniques to facilitate their use. Biomedical named entity recognition (bio-NER) is a form of NLP that identifies and categorizes biomedical terms in unstructured biomedical documents. Gene, protein, drug or disease are some common named entity classes considered in biomedical domain^2^. In recent years, bio-NER systems have been successfully used in a diverse set of applications such as bio-medical literature mining^3,4^, customer care, community websites or personal information management^5^.

Notwithstanding these achievements, the application of NER in the clinical domain still presents many challenges. Compared to the general NLP domain, determining the right boundaries of clinical named entities is a difficult task, since they are often multi-token terms with nested structures that include other entities inside them. In addition, the biomedical literature does not follow strict naming conventions. Instead, there are usually several ways to mention the same named entity and the use of symbols, digits and abbreviations is very common. This variability makes it difficult for matching-based unsupervised methods to work well in the clinical domain^6^. As a result, early bio-NER systems such as cTAKES^7^ or MetaMap^8^, which worked by matching text phrases with handcrafted dictionaries and rules, have been replaced or combined with supervised methods that learn to extract and categorize clinical terms from existing data. Thus, machine learning and hybrid based solutions like CLAMP^9^ and Bio-BERT^10^ have achieved state-of-the-art results in the field of bio-NER, although they heavily rely on annotated datasets to train and validate their models.

Over the last decade, several annotated corpora have been developed, including both manually annotated (known as gold standards) and automated or semi-automated annotated collections (silver standards)^11–14^. These corpora contain texts, extracted mainly from scientific articles and medical records, and their corresponding annotated named entities (e.g., diseases, body parts, treatments)^15^. Still, their availability is limited due to two main factors. First, annotating corpora manually is laborious and expensive, particularly so in the clinical domain in which medical expertise is required. Second, the access and exploitation of the source texts is often restricted by licensing terms and data privacy regulations, such as the Health Insurance Portability and Accountability Act (HIPAA)^1,14^. As a consequence, the available datasets are old (for instance, NCBI was last revised in August 27, 2013), require registration (as is the case of i2b2 dataset, now housed in the Department of Biomedical Informatics at Harvard Medical School) and/or force to obtain a human subject training certificate (e.g., for ShARe/CLEF, currently hosted by the MIT Lab for Computational Physiology).

As an alternative to the use of annotated datasets in the development of bio-NER tools, in this study we present a method based on the exploitation of omics data and network analysis. On the one hand, the increasing availability of omics data, such as genomic, proteomic, transcriptomic or metabolomic, resulting from improvements in the acquisition of molecular biology, represents an unprecedented resource for clinical researchers. Big data originating from biology are complemented with chemical and pharmacological data published by laboratories and regulatory agencies^16^. On the other hand, the emerging field of network medicine offers the tools of network science for interconnecting these data and discovering new insight about how diseases operate at the molecular level and how they are related to each other. Major projects such as DisGeNET^17^ and Hetionet^18^ have exploited this approach to obtain vast complex networks that enable researchers to formulate novel hypothesis on drug therapeutic action and drug adverse effects, and predict disease gene associations, among other applications^19^.

Previous studies have built phenotypic disease networks out of the named entities extracted from medical texts using bio-NER tools, and compared them with omics-based networks^20,21^. The results showed a very significant overlap between both types of networks, proving that shared terms (symptoms) indicate shared genes and proteins, for instance. Additionally, it was observed that disease networks obtained from medical texts tended to form clear, highly interconnected communities, which coincided significantly with the disease categories of classifications systems such as the disease ontology (DO) and the medical subject headings (MeSH)^22,23^. Given these precedents, our hypothesis is that the accuracy of a bio-NER tool can be measured by building a disease network from the extracted entities and calculating both its overlapping with omics networks and the coincidence of its communities with the categories of disease classification systems.

**Figure 1:**
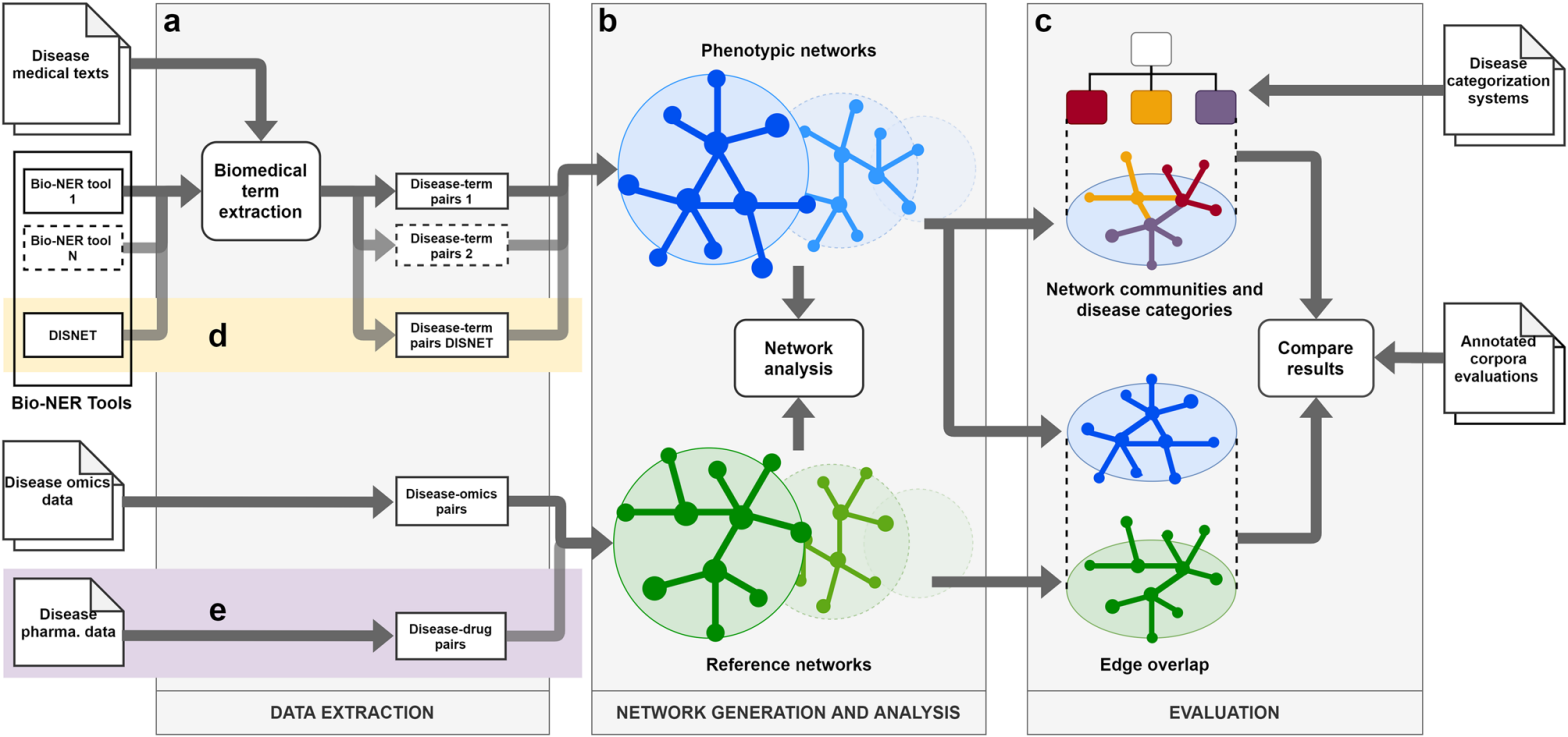
Experimental Design. (**a**) First, data are extracted from textual and omics sources; (**b**) next, networks are generated from the extracted data, and their main characteristics are analysed and compared; (**c**) finally, network-based criteria are applied to evaluate the accuracy of the bio-NER tool, and the results are compared with existing evaluations based on annotated corpora; (**d**) same method is applied to DISNET’s bio-NER system; and (**e**) the reference set is extended with pharmacologic data.

To test our hypothesis, we selected four bio-NER tools based on unsupervised (MetaMap^8^ and MetaMap Lite^24^), supervised (CLAMP^25^) and hybrid (BERN^26^) methods. First, we used each tool to extract medical terms from a dataset of Wikipedia and Mayo Clinic disease articles, and obtained their associated phenotypic disease networks by computing the similarity of the terms vector extracted for each disease. Second, we used the same approach to build omics disease networks from public available data sources (see Supplementary Table S6) and analyzed their overlapping with each phenotypic network. Third, we applied network analysis techniques to obtain the disease communities of the phenotypic networks and evaluated their coincidence with the top-level categories in MeSH, DO and International Classification of Diseases (ICD-10-CM). Finally, we compared the results to find the best performing tool and contrasted the outcome with classical evaluation approaches. Figure 1 illustrates the experimental design, which is thoroughly described in the “Methods” section.

Our study confirmed that the tools with highest accuracy when evaluated with annotated corpora generally rank first according to our method. In other words, we proved that our method performs similarly to strategies based on annotated corpora, without suffering from their limitations. We also demonstrated both the extensibility of this approach, by including the comparison with disease–disease networks obtained from pharmacological data, and its application to the evaluation of an alternative bio-NER tool.

## Results

### Characterization of disease networks

**Figure 2:**
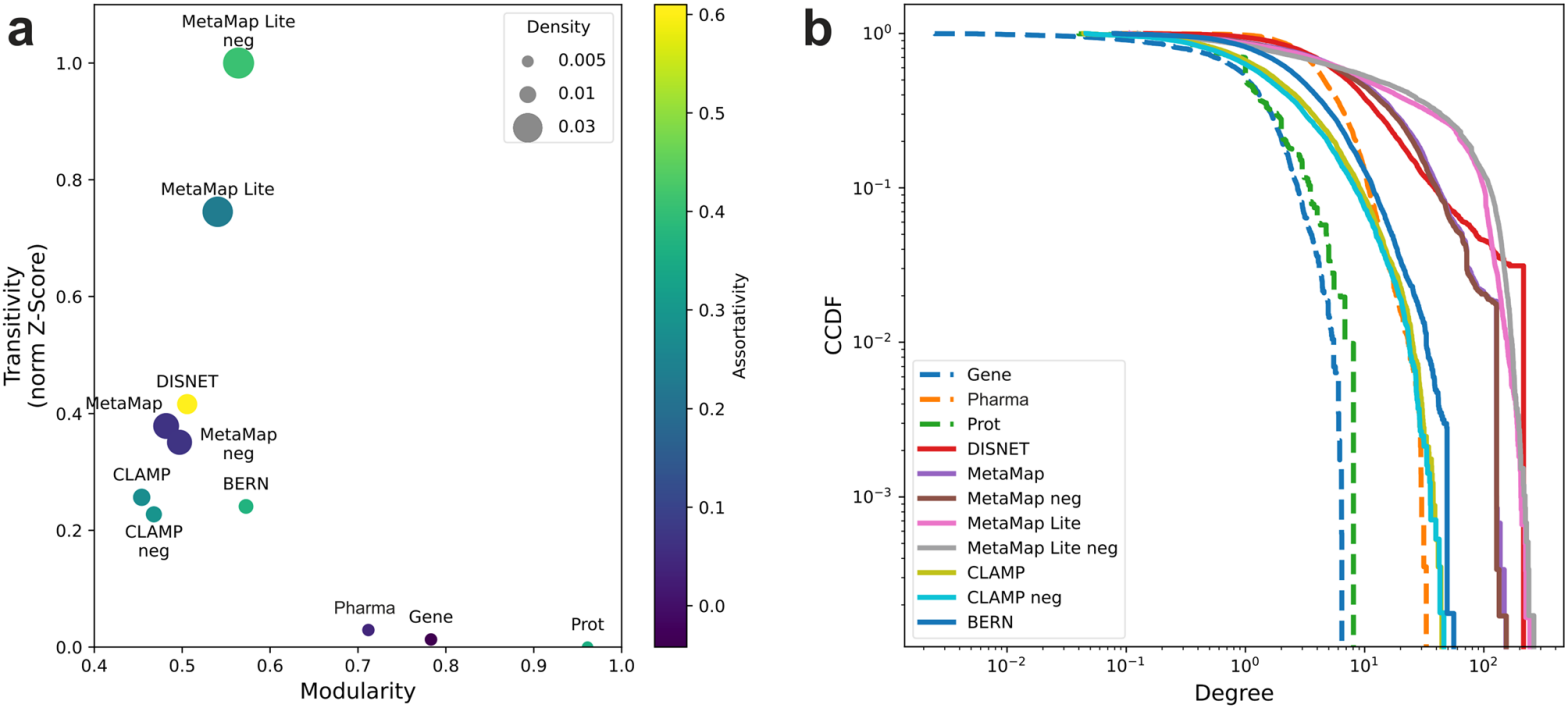
Comparison of network characteristics. (**a**) Location of the analysed networks in the normalized transitivity versus modularity plane. The size and the color of the bubbles represent the density and assortativity of the networks, respectively; (**b**) log–log plot of the degree CCDF of the networks.

Table 1 lists the main characteristics of both the phenotypic disease networks generated from the terms extracted with the bio-NER tools, and the reference disease networks obtained from genomic, proteomic and pharmacological data, as described in the “Methods” section. Figure 2 provides a visual representation of the results. In the case of phenotypic networks, while they present a similar number of nodes (ranging from 5054 to 6042), there is a significant variation in the number of edges (12,499 for BERN versus 595,110 for MetaMap Lite). While this implies that the tools are capable of extracting terms for approximately the same number of diseases, we found that the number of terms extracted per disease (and therefore, the connections between them) differs. For example, for *Larsen Syndrome*, BERN extracts 20 terms, compared to 42 for MetaMap. The density values, which range between 0.008 and 0.033, reflect this disparity and coincide with those of other phenotypic disease networks obtained from medical text mining^27^. For their part, the reference networks have a lower number of nodes, covering in the best case only 39.38% of the total diseases in the Wikipedia and Mayo Clinic article dataset (see “Methods” section), compared to a maximum of 84.01% for the phenotypic networks. This indicates a concentration of omics data on a limited set of diseases, while textual data cover a broader set. The density of the reference networks is also lower, with values around 0.005. Previous studies confirmed the low density of biological networks, arguing that they are generally sparsely connected, since this confers an evolutionary advantage for preserving robustness^28^.

**Table 1:** Characteristics of the extracted networks.

Network	Nodes	Edges	Density	Modularity	Transitivity (normalized z-score)	Assortativity
Genomic	1725	8,208	0.0055	0.783	0.013	− 0.042
Proteomic	713	1,169	0.0046	0.961	0.000	0.356
Pharmacologic	2832	21,817	0.0054	0.712	0.030	0.041
MetaMap	5903	411,282	0.0236	0.481	0.379	0.067
MetaMap (negation)	5900	386,967	0.0222	0.497	0.351	0.070
MetaMap Lite	6042	595,110	0.0326	0.540	0.745	0.230
MetaMap Lite (negation)	5872	585,465	0.0339	0.564	1.000	0.409
CLAMP	5676	171,382	0.0106	0.454	0.256	0.273
CLAMP (negation)	5627	144,936	0.0091	0.468	0.227	0.289
BERN	5683	124,999	0.0077	0.572	0.241	0.368
DISNET	5054	184,274	0.0144	0.505	0.416	0.610

Calculations of the transitivity, including the results of the normality tests, are available in the Supplementary Materials (see Supplementary Table S1).

**Table 2:** Bio-NER tools used in the study. MetaMap, MetaMap Lite and CLAMP provide configurable assertion detection (i.e., negation), hence the two performance values in the i2b2 2010 dataset.

Bio-NER Tool	Description	Performance (F1 Score)		
		i2b2 2010	SemEval 2014	NCBI disease
MetaMap	An open-source software program developed by the NLM for finding UMLS concepts in biomedical text using dictionary lookup	0.37, 0.38 (negation)	0.469	0.641
MetaMap Lite	A lightweight implementation of MetaMap, meant for applications that emphasize processing speed and ease of use	0.38, 0.45 (negation)	0.645	0.725
CLAMP	A clinical NLP toolkit that provides state-of-the-art NLP components and a user-friendly graphic user interface to build customized NLP pipelines. CLAMP uses various technologies, including machine learning-based methods and rule-based methods	0.857, 0.9398 (negation)	0.632	–
BERN (with Bio-BERT)	A neural biomedical named entity recognition and multi-type normalization tool. BERN uses the Bio-BERT NER models to tag genes/proteins, diseases, drugs/chemicals, and species	0.865	0.779	0.8936

As shown in Fig. 2a, the modularity of the reference networks is greater than in the networks obtained from texts. This denotes a greater tendency of omics networks to form communities, although the range of values obtained in the phenotypic networks (around 0.5) can also be considered as relatively high. Among them, the network associated with BERN presents the highest modularity. In contrast, the transitivity values of the phenotypic networks are generally higher than in the reference networks (see Supplementary Table S1 for more details). This suggests that, even though phenotypic networks have less tendency to cluster in communities, their communities are more densely connected internally, compared to biological networks. In the literature, networks with a 0.3 transitivity are considered highly transitive^29^. Our results show that the network associated with MetaMap Lite with negation detection presents the highest transitivity. Figure 2b displays the log–log plot of the degree complementary cumulative distribution function (CCDF) of the networks. For the phenotypic networks, their CCDFs show a less abrupt fall than those of the reference networks, especially genomics and proteomics. This indicates that the maximum degree in phenotypic networks is much higher than in biological networks, which is due to a greater interconnection of diseases through their symptoms, than through their associated genes or proteins. In other words, symptom-based connections are less specific than those based on genes or proteins^30,31^. Our results also show that phenotypic networks tend to be assortative, meaning that disease hubs tend to connect with each other. This property is also observed in social networks, for example^32^. In contrast, proteomic and genomic networks have low or negative assortativity, since their nodes tend to link to nodes with fewer interaction partners rather than to other hubs. Protein interaction networks and neural networks are documented examples of disassortative networks^32^. This confirms the greater specificity of biological bonds compared to phenotypic ones, previously observed with the degree distribution.

The pharmacological network, added in this study as an example of extension of the reference networks, presents mixed characteristics. On the one hand, its density, modularity and transitivity are similar to those of the omics networks. On the other hand, its topology (degree distribution and assortativity) is closer to phenotypic networks. This reflects that pharmacology is derived from both phenotypic and biological disease knowledge.

### Overlap of phenotypic and biological networks

Supplementary Table S2 lists the number of common nodes (diseases) and edges between each phenotypic network associated with a bio-NER tool, and the reference networks, as well as the z-scores obtained when comparing the values with those expected at random, and the *p *values corresponding to the Shapiro–Wilk test (see “Methods” section). Phenotypic networks share a similar number of nodes with reference networks. For example, the network associated with MetaMap Lite has 1506 nodes in common with the genomic network (87.30%), compared to 1470 (85.22%) of CLAMP and 1487 (86.21%) of BERN. This result was expected since, as presented in the previous section, the networks obtained from bio-NER tools have a similar number of nodes. In the same way, given that they have an uneven number of links, it was also expected that the number of overlapping links would be different, as reflected in the results. Thus, while MetaMap Lite shares 759 links with the genomic network (9.25%), MetaMap only shares 437 (5.32%). In all cases, the z-score, which indicates the significance of this overlap with respect to the random case, is higher for the phenotypic network associated with BERN. CLAMP performs second best, followed by MetaMap Lite and MetaMap. Only in the case of MetaMap Lite, the network obtained with negation detection presents a clearly superior performance than without this function.

Supplementary Table S3 contains the results for the overlap of the phenotypic networks with all the reference networks simultaneously. In this case the number of shared nodes and links is drastically reduced. Only around 340 diseases in phenotypic networks are present in the genomic, proteomic and pharmacological networks, and the number of overlapping edges ranges from 18 to 33. The z-score confirms the ranking obtained when using the reference networks separately, which suggests that the type of reference network used to measure the overlap with the phenotypic networks has little influence. Taking into account this result and that the size of the combined network would limit the validation of bio-NER to a reduced set of diseases, we discarded this test in favor of the overlapping with individual omics networks.

### Coincidence of communities in phenotypic network with disease categories

Supplementary Table S4 shows the number of communities obtained with the Louvain method for each phenotypic network (see “Methods” section), as well as their ratio of coincidence with the top-level categories in MeSH, DO and ICD-10-CM, the z-scores computed by comparing the values with those obtained for random networks, and corresponding p-values of the Shapiro–Wilk test. The evaluation of bio-NER tools assessed with this method is generally consistent across disease classification systems, and also with that obtained by measuring the edge overlap with the reference networks.

**Figure 3:**
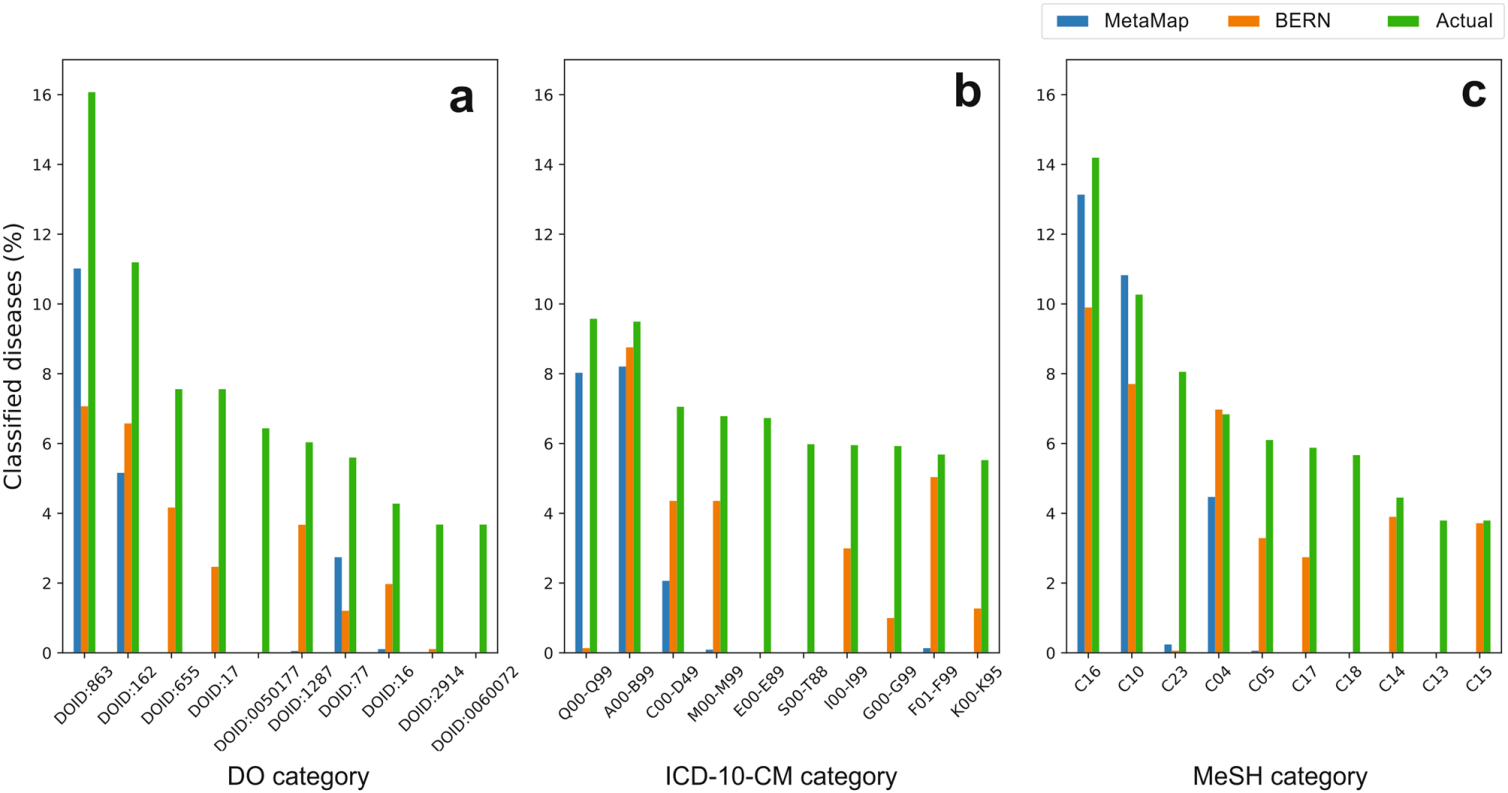
Coincidence of network communities with disease categories. The bar plots show the proportion of diseases associated with the 10 largest first-level categories in the DO (**a**), ICD-10-CM (**b**) and MeSH (**c**) classification systems, compared with the proportion obtained for the best performer (BERN) and worst performer (MetaMap).

Figure 3 shows the percentage of diseases classified in the 10 largest top-level categories of DO (Fig. 3a), MeSH (Fig. 3b) and ICD-10-CM (Fig. 3c), for the best performer (BERN) and worst performer (MetaMap), as a result of the previous analysis. For reference, it also displays the actual percentage of diseases that belong to those categories in each classification system. E.g., out of a total of 2501 diseases in the dataset mapped to a DO concept, 402 (16.07%) have the category DO 863 (diseases of the nervous system). We observe that the communities of the phenotypic networks present a similar degree of coincidence for the equivalent categories in the different classification systems. In the case of BERN, we find greater coincidences in the categories MeSH C04, DO 162 and ICD-10-CM C00-D49 (neoplasms/cancer); MeSH C05, DO 17 and ICD-10-CM M00-M99 (diseases of musculoskeletal system); and MeSH C14, DO 1287 and ICD-10-CM I00-I99 (cardiovascular diseases/diseases of circulatory system). For its part, MetaMap presents greater coincidences in MeSH C10, DO 863 and ICD-10-CM G00-G99 (diseases of the nervous system). This suggests that bio-NER tools are capable of extracting terms, and ultimately relationships between diseases, consistently with classifications of diseases, as described in the literature^22, 23^.

### Comparison with gold-standard based evaluation

**Figure 4:**
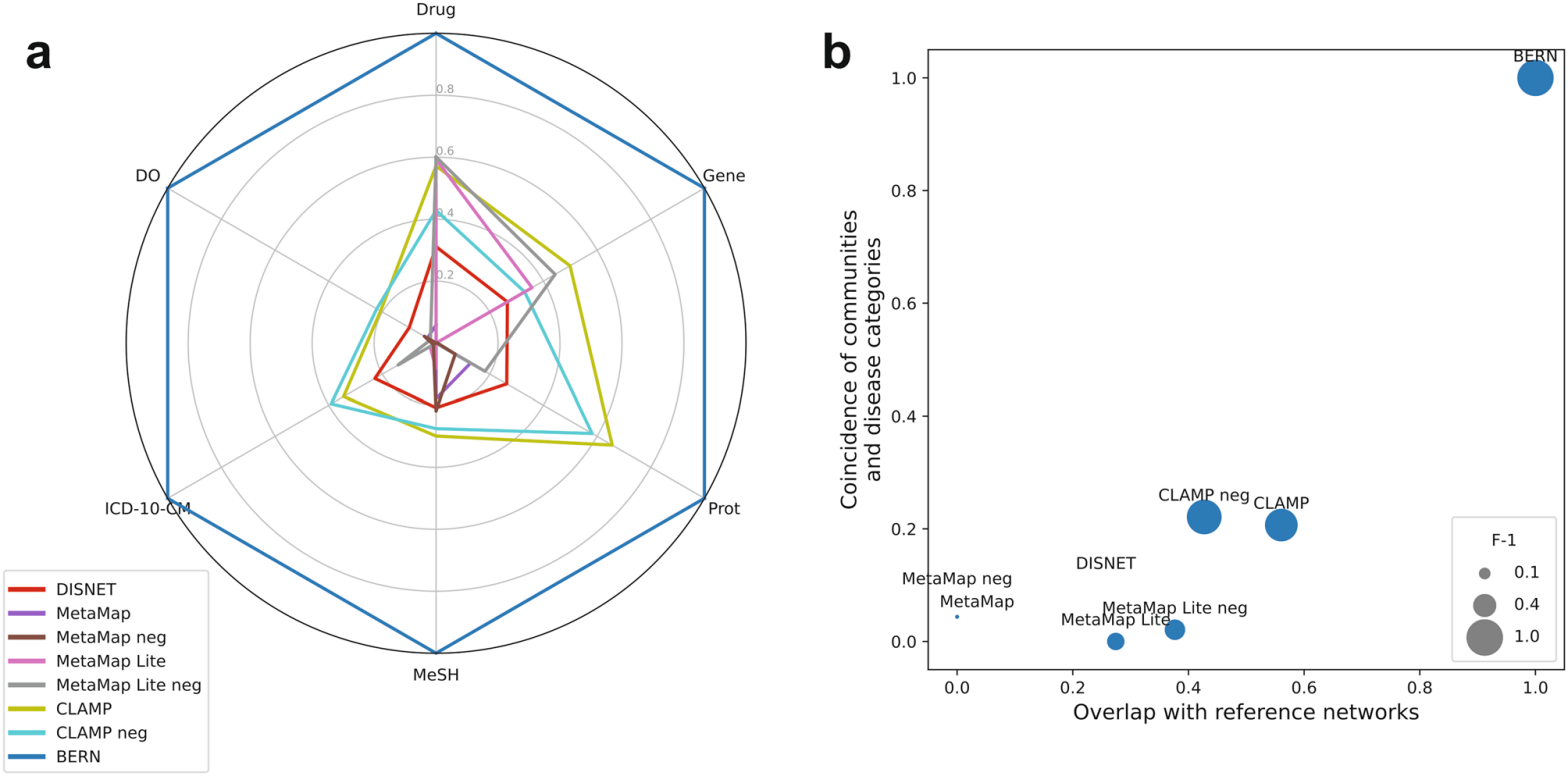
Evaluation of the bio-NER accuracy according to the proposed model. (**a**) Results of the network overlapping and community coincidence tests and (**b**) normalized average results for the two tests, compared with the normalized average F-1 score of the bio-NER tools obtained from gold-standard based evaluations.

The spider web chart in Fig. 4a summarizes visually the results of the tests described in the previous sections. The network associated with BERN performs best both in the overlap with the reference networks and in the coincidence of its communities with the disease categories. Overall, the two CLAMP variations (with and without negation detection) have the second-best performance. Only in the overlap with the pharmacological network, the results of MetaMap Lite (with and without negation) are similar to those of CLAMP. MetaMap obtains comparatively the worst results, except in the coincidence of communities with MeSH categories, where MetaMap Lite performs worse.

In order to compare the global results of both tests, Fig. 4b represents their normalized mean values. According to our proposed evaluation of bio-NER tools, the better the results in the tests (that is, the further up and right in the chart), the greater the accuracy of the tool. To validate our approach, we contrasted our evaluation results with those obtained through traditional methods based on annotated corpora. In Fig. 3b, the area of the bubble represents the normalized mean F-1 value of the tool (see Table 2). We observe that there is a notable correlation between the position and the size of the plots, with BERN outperforming the other tools, CLAMP ranking second, followed by MetaMap Lite and MetaMap.

Our assessment coincides even for the variations within the same tool. Both MetaMap and MetaMap Lite perform better when negation detection is enabled. Only for CLAMP, we observed a difference with respect to the F-1-based ranking. Its accuracy is higher with negation detection, according to the evaluation performed with the i2b2 dataset (the only data available for this case), but our method gives a slightly greater accuracy to the variation without detection.

### Application to DISNET

To test the application of our evaluation method to an alternative bio-NER tool, we performed the same tests with DISNET's text extraction system, which is built on top of MetaMap with an additional dictionary-based validation of terms. The tables and figures in the previous sections include the results for this tool. According to our evaluation, the accuracy of DISNET's bio-NER is higher than that of MetaMap alone. This was expected, since the validation system eliminates false positives caused by the ambiguity of the terms detected by MetaMap^8,33^. DISNET has an accuracy comparable to that of MetaMap Lite, but noticeably worse than solutions based on more advanced NER methods such as CLAMP or BERN.

## Discussion

In this study, we hypothesize that the increasingly available omics data can be used in combination with network analysis to evaluate bio-NER tools, as an alternative to traditional methods based on annotated corpora. To demonstrate our hypothesis, we first built a dataset of medical texts associated with diseases from public textual sources and used 4 bio-NER tools with known F-1 value to extract their clinical terms. Next, by computing the pairwise similarity between diseases based on the extracted terms, we generated the disease-disease phenotypic network corresponding to each tool. Additionally, we collected publicly available data on disease-gene and disease-protein associations to build reference omics networks, following the same method. The analysis of the networks, illustrated in Fig. 2, shows that their characteristics coincide with those of other networks generated in a similar way, confirming the validity of our process up to this point.

In a first test, we measured the overlapping of the phenotypic network of each bio-NER tool with the omics networks. In a second test, we evaluated the coincidence of the communities of the phenotypic networks with the top-level categories of various classification systems. The obtained results show that a better performance of the bio-NER tool in the network overlapping and community coincidence tests is associated with a greater precision of the tool when it is evaluated using gold-standards. Therefore, as proposed in our hypothesis, a metric composed of the results of both network-based tests can replace the F-1 obtained through validation with annotated corpora, as illustrated in Fig. 4b.

Since annotated datasets are generally scarce, limited access and outdated, our method offers researchers an alternative based on more abundant, accessible and updated omics data. Furthermore, our approach allows other sources to easily be incorporated, as we demonstrated when using disease-drug associations. However, our solution has some limitations. First, although it makes it possible to clearly differentiate the accuracy of two different tools, it is less precise when comparing variations within the same tool, as we observed in the case of CLAMP with and without negation detection. Second, using this method requires disease-associated text sets, such as the Wikipedia and Mayo Clinic articles used in the study. Clinical texts such as EMRs, where several disorders might be discussed simultaneously, are not suitable. Last, our method only measures the accuracy of bio-NER tools, without evaluating other important aspects such as their speed or their usability.

To improve the precision of our method, in the case of the overlap with reference networks, we propose the exploitation of new sources (e.g., transcriptomics, metabolomics, epigenomics) to build a more complete set of reference networks. Regarding the coincidence of the communities with the disease categories, on the one hand it is necessary to evaluate whether alternative community detection methods offer better results. And on the other hand, we recommend studying the different hierarchical levels of the classification systems, in order to find the most appropriate level for this test. Finally, by extending the study to more bio-NER tools with known accuracy (e.g., from NER challenges in this area), it should be possible to determine which reference networks or classification systems in particular offer results closer to the reference ones, and favor their use to improve the efficiency of our method.

## Methods

### Experimental design

The goal of our research is to provide an alternative to the use of annotated corpora for the evaluation of bio-NER tools. Based on the previous work presented in the introduction, our hypothesis is that the accuracy of a bio-NER tool can be assessed through the analysis of the disease network generated from the extracted terms, including its overlap with omics networks and the coincidence of its communities with the categories of disease classification systems.

Figure 1 describes the experimental design to demonstrate our hypothesis. We first used several bio-NER tools to extract disease-term pairs from a dataset of medical articles, and mined omics sources to obtain disease-gene and disease-protein pairs (Fig. 1a). Next, we built the phenotypic and reference disease–disease networks out of the disease-term pairs and disease-omics pairs, respectively, and analysed their characteristics (Fig. 1b). Finally, we evaluated the overlap between the phenotypic and omics networks as well as the coincidence of the phenotypic network communities with different disease categorizations, and contrasted the results with the bio-NER tool evaluations obtained with annotated datasets (Fig. 1c). Additionally, we demonstrated the applicability of our method to the assessment of an alternative bio-NER (Fig. 1d) and its extensibility by expanding the set of reference networks with pharmacological data (Fig. 1e).

### Bio-NER tools

In our study, we used four bio-NER tools: MetaMap^8^, MetaMap Lite^24^, CLAMP^25^ and BERN^26^. We selected these tools based on three aspects: (1) they are publicly available; (2) they use different bio-NER approaches (rule-based, dictionary-based, ML and hybrid); and (3) their accuracy has been evaluated against different gold standards. These criteria ensure the reproducibility, generalizability and evaluability (respectively) of our method. Table 2 shows a brief description for each tool and its performance evaluated against the i2b2 2010^12^, SemEval 2014^34^ and NCBI^11^ datasets. For more detailed information on the tools, including the version and configuration used in the study, see Supplementary Table S5.

### Disease–disease networks from text datasets

For the extraction of medical terms through the bio-NER tools, we used a dataset consisting of excerpts of 7500 Wikipedia articles and 620 Mayo Clinic articles, obtained between 2019 and 2020 as part of the DISNET project^35^. Each article is associated with a single disease, and there may be more than one article for the same disease. As a whole, the dataset contains texts for 7192 diseases, with a total of 3,330,001 words and an average of 463.01 words per disease (standard deviation = 56.57). We used the Crosswalk Vocabulary API of the Unified Medical Language System (UMLS) to map the diseases by their identifiers in different terminologies^36^. See Supplementary Table S6 for more details.

We processed the dataset with each bio-NER tool and extracted the named entities associated with every disease. Next, we computed the pairwise similarities between diseases expressed as vectors of the extracted terms, using the Jaccard distance^37^. Finally, we built the disease-disease networks, in which two nodes (diseases) are connected with an edge weighted by the similarity of their extracted terms. To limit the size of the networks, only pairs with a similarity above the 95th percentile were considered. For the tools that support negation detection (MetaMap, MetaMap Lite and CLAMP), we obtained two networks, with and without this option.

### Disease–disease networks from biological sources

Data of gene-disease associations were obtained from DisGeNET^17^. For its part, the implications of proteins in diseases were extracted from Uniprot^38^. In order to demonstrate the aggregation of new sources to our system, we also incorporated data of disease-drug associations extracted from the Stanford Network Analysis Project^39^. Again, we used UMLS to cross-map the disease identifiers in the different sources. As in the case of text-extracted terms, we built the genomic, proteomic and pharmacological disease–disease weighted networks from the pairwise similarity of their genes, proteins and drugs, respectively. Due to the greater specificity of omics data, the number of obtained pairs was much lower than with the text terms, so they were not filtered. See Supplementary Table S6 for more details.

### Network characterization

As a previous step to the application of our method in the evaluation of the bio-NER tools, we performed an analysis of the characteristics of their associated networks using the *NetworkX* Python library^40^.

First, we measured three dimensions of the network structure: density, modularity, and transitivity. The network density is defined as the number of existing relationships relative to the possible number. For its part, the modularity measures the degree to which the network tends to segregate into relatively independent groups. It is computed as the fraction of the edges that fall within the groups, minus the expected fraction if edges were distributed at random. Biological networks have a significantly higher modularity compared to random networks, which proves their modular nature^41^. However, it has been shown that modularity suffers a resolution limit and, therefore, it is unable to detect small communities. On the other hand, the transitivity of a network is the relative proportion of triangles among all connected triads it contains. It can be interpreted as the probability of finding a direct connection between two nodes having a common neighbor. In general, high transitivity allows obtaining a community structure. However, high transitivity is not a prerequisite to the existence of a strong community structure^42^.

Next, we obtained data on the network topology, including the degree distribution and assortativity. The degree distribution P(k) of a network is the probability that a randomly chosen node has k connections (or neighbours). In most complex networks (including biological networks), the degree distribution is highly asymmetric due to the presence of a small number of highly connected nodes (hubs)^43,44^. To compare the degree distributions of the networks, we computed the complementary cumulative distribution function (CCDF), also known as tail distribution^45^. If the resulting plot of one distribution falls above the other, we may conclude that the upper one has a heavier tail (i.e., decays slower) than the lower. The assortativity is another measure related to the network topology, and indicates the preference for a network's nodes to attach to others that are similar in some way. Thus, a network is called assortative (i.e., its assortativity ranges from 0 to 1) if the vertices with higher degree have the tendency to connect with other vertices that also have high degree of connectivity. If the vertices with higher degree have the tendency to connect with other vertices with low degree, then the network is called disassortative (i.e., the assortativity is between 0 and − 1).

Finally, we compared the results obtained for the phenotypic and reference disease-disease networks with each other and with the existing literature.

### Network overlapping

For each bio-NER tool, we obtained the edges shared between its associated disease network and the reference networks, using *NetworkX*. Next, we compared the number of observed overlapping edges to what would be expected with random networks. The Statistical Analysis section describes the statistical methods used in more detail.

### Community detection

As explained in the introduction, several studies have reported significant overlaps between communities in phenotypic networks and disease categories^22,23^. To replicate this analysis, we first obtained the disease categories of first hierarchical level from the MeSH, ICD-10-CM, and DO classification systems. MeSH descriptors were downloaded from the NLM site. Only categories of type C (Diseases) and F03 (Mental Disorders) were considered. The ICD-10-CM code descriptions were downloaded from the website of the Centers for Medicare and Medicaid Services, and concepts of the DO were obtained from the code repository of the project. Finally, UMLS and DO mappings were used to associate the categories with the diseases in the networks (see Supplementary Table S6).

To detect the communities in the disease networks, we used Louvain's method, which optimizes modularity as the algorithm progresses^46^. First, for each disease network associated with a bio-NER tool, we obtained the best partition using the *Community* library from the *Python-Louvain* Python package^47^. Then, for each community obtained, we computed its associated disease category in each classification system (i.e., the most frequent among its diseases) and the proportion of community members that belonged to that category. The result indicated the ratio of coincidence of the network communities with the disease categories. Finally, as in the case of network overlaps, we compared the value obtained with that expected at random (see Statistical Analysis).

### Comparison with DISNET extraction tool (TVP)

The DISNET database integrates phenotypic and genetic-biological characteristics of diseases and information on drugs from several expert-curated sources and unstructured textual sources^35^. Phenotypic data is extracted from Wikipedia, PubMed, and Mayo Clinic texts, using MetaMap and a validation system called term validation process (TVP). The TVP aims to eliminate false positives detected by MetaMap and increase the precision of the results. It could be thought of as a dictionary-based extension to MetaMap. Evaluating this extraction mechanism against an annotated dataset shows a performance improvement over MetaMap alone^33^. In order to demonstrate the application of our approach to a new bio-NER tool, we used the DISNET extraction system to obtain the terms of our dataset and performed the same analyses as for the rest of the tools.

### Statistical analysis

To evaluate the statistical significance of the network transitivity, the overlap of the phenotypic and reference layers, and the coincidence of the network communities with the disease categories, we obtained for each bio-NER tool a network with the same number of randomly connected nodes and performed the same analysis. We repeated the randomization process 1000 times and recorded the results to obtain a distribution that served as a null model. We verified the normality of this distribution through the Shapiro–Wilk test (i.e., *p* value > 0.05 implies that it is normal). Finally, we calculated the z-scores of the results observed in the original networks with respect to the null model. A higher magnitude of the z-score (either positive or negative) indicates a greater statistical significance of the result. When a better comparability of the z-scores was needed, we used min–max normalization to scale their range in [0, 1]. The *p *values of the Shapiro–Wilk normality tests and the z-scores are included in the Supplementary Materials.

## Supplementary Information

Supplementary Tables.

## Data Availability

All data needed to evaluate the conclusions in the paper are present in the paper and/or the Supplementary Information. The datasets generated during and/or analysed during the current study are available from the corresponding author on reasonable request.
