# Supplementary material for: Leveraging network analysis to evaluate biomedical named entity recognition tools
Source: Sci Rep. 2021 Jun 29;11:13537. doi: 10.1038/s41598-021-93018-w (PMC8242017; doi:10.1038/s41598-021-93018-w)
Supplement: Supplementary file 1 — Supplementary Tables. [file 41598_2021_93018_MOESM1_ESM.docx]

**Supplementary Information**

**Leveraging network analysis to evaluate biomedical named entity recognition tools**

Eduardo P. García del Valle^1*^, Gerardo Lagunes García^1,2^, Lucía Prieto Santamaría^2^, Massimiliano Zanin^3^, Ernestina Menasalvas Ruiz^1,2^, Alejandro Rodríguez-González^1,2^.

^1^ ETS de Ingenieros Informáticos. Universidad Politécnica de Madrid. Boadilla del Monte, Madrid, Spain.

^2^ Centro de Tecnología Biomédica, ETS Ingenieros Informáticos. Universidad Politécnica de Madrid. Pozuelo de Alarcón, Madrid, Spain.

^3^ Instituto de Física Interdisciplinar y Sistemas Complejos IFISC (CSIC-UIB), Campus UIB, Palma de Mallorca, Spain.

*Correspondence should be addressed to Eduardo P. García del Valle. (ep.garcia@alumnos.upm.es).

**Table S1.** Network transitivity. The z-score is that of the transitivity computed for each network, with respect to the expected at random. The p-value corresponds to the Shapiro-Wilk normality test of the random distribution.

| Network | Transitivity | Z-score | p-value (Shapiro-Wilk test) |
| --- | --- | --- | --- |
| Genomic | 0.232 | 573.642 | 0.074 |
| Proteomic | 0.603 | 304.219 | 0.393 |
| Pharmacologic | 0.229 | 912.840 | 0.051 |
| MetaMap | 0.277 | 8,158.019 | 0.722 |
| MetaMap (negation) | 0.280 | 7,326.089 | 0.358 |
| MetaMap Lite | 0.503 | 17,697.776 | 0.678 |
| MetaMap Lite (negation) | 0.602 | 18,666.394 | 0.974 |
| CLAMP | 0.304 | 5,657.900 | 0.993 |
| CLAMP (negation) | 0.314 | 5,055.710 | 0.693 |
| BERN | 0.324 | 4,362.481 | 0.066 |
| DISNET | 0.543 | 11,122.057 | 0.282 |

**Table S2.** Overlap of phenotypic networks associated with the bio-NER tools and the reference networks. The nodes and edges columns contain the number of shared nodes and edges, respectively, between each phenotypic and reference network. The z-score is that of the number of shared edges, with respect to the expected at random. The p-value corresponds to the Shapiro-Wilk normality test of the random distribution.

| Network | Overlap | Nodes | Edges | Z-Score | p-value (Shapiro-Wilk test) |
| --- | --- | --- | --- | --- | --- |
| Metamap | Genomic | 1,469 | 437 | 40.528 | 0.269 |
|  | Proteomic | 601 | 138 | 43.961 | 0.052 |
|  | Pharmacologic | 2,434 | 739 | 38.988 | 0.575 |
| MetaMap (negation) | Genomic | 1,462 | 407 | 36.184 | 0.496 |
|  | Proteomic | 601 | 125 | 38.860 | 0.051 |
|  | Pharmacologic | 2,425 | 677 | 38.757 | 0.964 |
| MetaMap Lite | Genomic | 1,506 | 759 | 66.136 | 0.460 |
|  | Proteomic | 603 | 141 | 33.745 | 0.393 |
|  | Pharmacologic | 2,425 | 677 | 38.757 | 0.964 |
| MetaMap Lite (negation) | Genomic | 1,485 | 868 | 77.891 | 0.420 |
|  | Proteomic | 602 | 179 | 44.447 | 0.052 |
|  | Pharmacologic | 2,437 | 1392 | 73.136 | 0.450 |
| CLAMP | Genomic | 1,470 | 502 | 95.677 | 0.499 |
|  | Proteomic | 593 | 172 | 79.809 | 0.086 |
|  | Pharmacologic | 2,395 | 664 | 59.019 | 0.595 |
| CLAMP (negation) | Genomic | 1,459 | 404 | 65.740 | 0.420 |
|  | Proteomic | 589 | 144 | 66.223 | 0.050 |
|  | Pharmacologic | 2,375 | 548 | 61.282 | 0.500 |
| BERN | Genomic | 1,487 | 608 | 112.594 | 0.208 |
|  | Proteomic | 610 | 189 | 114.108 | 0.050 |
|  | Pharmacologic | 2,420 | 731 | 81.595 | 0.443 |
| DISNET | Genomic | 1,391 | 433 | 58.032 | 0.783 |
|  | Proteomic | 574 | 126 | 54.294 | 0.051 |
|  | Pharmacologic | 2,355 | 662 | 54.679 | 0.772 |

**Table S3.** Overlap of phenotypic networks associated to the bio-NER tools and the combined reference networks. The nodes and edges columns contain the number of shared nodes and edges, respectively, between each phenotypic network and all reference networks combined. The z-score is that of the number of shared edges, with respect to the expected at random. The p-value corresponds to the Shapiro-Wilk normality test of the random distribution.

| Network | Nodes | Edges | Z-score | p-value (Shapiro-Wilk test) |
| --- | --- | --- | --- | --- |
| MetaMap | 340 | 24 | 22.877 | 0.971 |
| MetaMap (negation) | 340 | 23 | 22.236 | 0.301 |
| MetaMap Lite | 342 | 18 | 17.553 | 0.990 |
| MetaMap Lite (negation) | 342 | 27 | 23.373 | 0.727 |
| CLAMP | 344 | 33 | 48.803 | 0.435 |
| CLAMP (negation) | 343 | 30 | 47.767 | 0.659 |
| BERN | 347 | 33 | 59.002 | 0.194 |
| DISNET | 328 | 18 | 27.379 | 0.179 |

**Table S4.** Coincidence of the communities detected in the phenotypic networks and the top-level disease categories of MeSH, ICD-10-CM and DO. The ratio column represents the proportion of community members that match the expected category. The z-score is that of coincidence ratio, with respect to the expected at random. The p-value corresponds to the Shapiro-Wilk normality test of the random distribution.

| Network | Commu-nities | Coincidence with MeSH top categories | | | Coincidence with ICD-10-CM top categories | | | Coincidence with DO top categories | | |
| --- | --- | --- | --- | --- | --- | --- | --- | --- | --- | --- |
|  |  | Ratio | Z-score | p-value (S-W test) | Ratio | Z-score | p-value (S-W test) | Ratio | Z-score | p-value (S-W test) |
| MetaMap | 29 | 0.293 | 98.849 | 0.050 | 0.175 | 26.068 | 0.050 | 0.189 | 28.782 | 0.050 |
| MetaMap (negation) | 29 | 0.305 | 46.254 | 0.054 | 0.190 | 34.399 | 0.732 | 0.184 | 21.957 | 0.188 |
| MetaMap Lite | 22 | 0.273 | 21.932 | 0.143 | 0.158 | 17.507 | 0.254 | 0.196 | 34.325 | 0.050 |
| MetaMap Lite (negation) | 41 | 0.249 | 7.972 | 0.823 | 0.155 | 25.889 | 0.032 | 0.208 | 62.213 | 0.086 |
| CLAMP | 25 | 0.364 | 174.241 | 0.667 | 0.275 | 87.099 | 0.449 | 0.252 | 80.689 | 0.051 |
| CLAMP (negation) | 23 | 0.352 | 73.990 | 0.051 | 0.298 | 87.976 | 0.198 | 0.259 | 77.392 | 0.050 |
| BERN | 21 | 0.464 | 259.626 | 0.062 | 0.348 | 63.778 | 0.586 | 0.351 | 273.470 | 0.054 |
| DISNET | 9 | 0.331 | 43.509 | 0.070 | 0.304 | 63.957 | 0.050 | 0.207 | 29.488 | 0.883 |

**Table S5.** Bio-NER tool details. Supplementary information to Table 2.

| Bio-NER Tools | Website | Access requirements | Version / access date | Configuration | Comments |
| --- | --- | --- | --- | --- | --- |
| MetaMap | https://metamap.nlm.nih.gov | UMLS license (Free) | 2018AB | -AGpIy+ -J acab, anab, comd, cgab, dsyn, emod, fndg, inpo, mobd, neop, patf, sosy, virs, bact [--negex] | Restricted to semantic types included as “problems” in the i2b2 challenge (*12*).  Negex option used for negation detection. |
| MetaMap Lite | https://metamap.nlm.nih.gov/MetaMapLite.shtml | UMLS license (Free) | 2018AB | semantictypes: [acab, anab, comd, cgab, dsyn, emod, fndg, inpo, mobd, neop, patf, sosy, virs, bact] | Restricted to semantic types included as “problems” in the i2b2 challenge (*12*).  Default negation detector class. (gov.nih.nlm.nls.metamap.lite.NegEx) |
| CLAMP | https://clamp.uth.edu | Request to authors and UMLS license (Free) | CMD 1.6.1 | Default (General Clinical Concept Extraction) | Only terms identified with semantic type “problem” were considered. |
| BERN (with BioBERT) | https://bern.korea.ac.kr | Request to authors (Free) | May, 2020 | Default | Only terms identified with object type “disease” were considered. |

**Table S6.** Data sources.

| Source | Website | Access requirements | Version / access date | Data |
| --- | --- | --- | --- | --- |
| Wikipedia | https://www.wikipedia.org | Free | January, 2020 | Encyclopedic articles on diseases. |
| Mayo Clinic | https://www.mayoclinic.org | Free | January, 2020 | Encyclopedic articles on diseases. |
| DisGeNET | https://www.disgenet.org | Free | v6.0 | Disease-gene associations. |
| UniProt | https://www.uniprot.org | Free | May, 2020 | Disease-protein associations. |
| Stanford Network Analysis Project | http://snap.stanford.edu | Free | May, 2020 | Disease-drug associations. |
| DO | https://disease-ontology.org | Free | tag v2018-03-02 | Disease classification.  Disease cross-mapping. |
| MeSH | https://www.nlm.nih.gov/databases/download/mesh.html | Free | 2018 | Disease classification. |
| ICD-10-CM | https://www.cms.gov/Medicare/Coding/ICD10 | Free | 2018 | Disease classification. |
| UMLS | https://documentation.uts.nlm.nih.gov/rest/home.html | UMLS License (Free) | 2018AB | Disease cross-mapping. |
